# Dissecting Driver Behaviors Under Cognitive, Emotional, Sensorimotor, and Mixed Stressors

**DOI:** 10.1038/srep25651

**Published:** 2016-05-12

**Authors:** I. Pavlidis, M. Dcosta, S. Taamneh, M. Manser, T. Ferris, R. Wunderlich, E. Akleman, P. Tsiamyrtzis

**Affiliations:** 1 Computational Physiology Laboratory, University of Houston, Houston, Texas 77204, USA; 2 Texas A&M Transportation Institute, Texas A & M University System, College Station, Texas 77843, USA; 3 Industrial and Systems Engineering, Texas A & M University, College Station, Texas 77843, USA; 4 Visualization Department, Texas A & M University, College Station, Texas 77843, USA; 5 Department of Statistics, Athens University of Economics and Business, Athens 104 34, Greece

## Abstract

In a simulation experiment we studied the effects of cognitive, emotional, sensorimotor, and mixed stressors on driver arousal and performance with respect to (wrt) baseline. In a sample of *n* = 59 drivers, balanced in terms of age and gender, we found that all stressors incurred significant increases in mean sympathetic arousal accompanied by significant increases in mean absolute steering. The latter, translated to significantly larger range of lane departures only in the case of sensorimotor and mixed stressors, indicating more dangerous driving wrt baseline. In the case of cognitive or emotional stressors, often a smaller range of lane departures was observed, indicating safer driving wrt baseline. This paradox suggests an effective coping mechanism at work, which compensates erroneous reactions precipitated by cognitive or emotional conflict. This mechanisms’ grip slips, however, when the feedback loop is intermittently severed by sensorimotor distractions. Interestingly, mixed stressors did not affect crash rates in startling events, suggesting that the coping mechanism’s compensation time scale is above the range of neurophysiological latency.

From the time of Henry Ford, the car has been powering the world economy and defining the modern way of life^1^. Every morning millions of cars take to the highways transporting commuters from their suburban homes to metropolitan business centers; late in the afternoon the same cars transport the same people back to their places. In these daily commutes, thousands of car accidents incur a significant material and human cost while reducing productivity. It is telling that rush hour traffic reports on radio are a staple of metropolitan areas. The focus of these short segments is on the accidents that took place and how these accidents are affecting traffic flow.

Highway driving is a challenging task. Human physiology responds to challenges through sympathetic arousal. To cope successfully with the task at hand, a measured sympathetic response is required - too little or too much is an invitation to failure^2^,^3^. In some instances elevated arousal is precipitated by a secondary task, which deprives resources from the main task; these cases are suspected to be the most prevalent cause of commuter traffic accidents^4^. Texting is a well-known example of a secondary task antagonistic to driving; it is a sensorimotor stressor, where the driver needs to move her/his eyes and one hand between the car’s controls and the smartphone all the time. Most other types of antagonistic stressors are cognitive or emotional in nature^5^. There has been little work about the distracting effect of each stressor category. Typically, the studies reported in the literature refer indirectly either to a sensorimotor stressor or to an unspecified mix of stressors^6^. The problem stems from poor abstraction, with most experimental designs centered on devices rather than stressor types. A driver may employ no device, appearing to be concentrated on the driving task, and still be under a hidden stressor (i.e., cognitive or emotional) that is potentially as distracting as an apparent stressor (e.g., texting).

The lack of clearly abstracted studies on driving distractions has as a result partially informed regulations; there are traffic laws that ban texting while driving but no consideration has been given to driving under cognitive or emotional distress. Should cognitive and emotional distractions prove damaging to driving behaviors, the problem is how do authorities regulate this? It is difficult to prove in a court of law somebody’s inner thoughts or feelings, the intensity of which many times the subject herself/himself underestimates. This brings to the fore the need for methods to sense the sympathetic and driving effect of various types of stressors, enabling timely orthotic actions.

Here we report results on a driving simulator experiment where subjects operated under normal and stressful conditions; the stressful conditions featured four types of stressors - cognitive, emotional, sensorimotor, and mixed. We delivered the cognitive and emotional stressors through appropriate oral questionnaires. We delivered the sensorimotor stressor in the form of texting while driving. For mixed stressors or absence thereof, subject behaviors were measured at two time scales - sustained engagement without exogenous surprises versus reactions to startling events.

We used instantaneous perspiration at the perinasal area as proxy of the subject’s sympathetic state^7^, thus forming the study’s explanatory variable. We used instantaneous steering angle and maximum right-side/left-side lane departures as proxies of the subject’s steering and driving performance, respectively, thus forming the study’s response variables. The perinasal perspiration signal was extracted through facial thermal imaging according to the method reported by Shastri *et al.*^8^. The steering angle and lane departure signals were recorded by the driving simulator.

The response variables were abstracted per the model introduced by Pavlidis *et al.*^3^. The purpose of this model is to establish causality links between explanatory and response variables by differentiating between reactionary response, driven purely by sympathetic arousal, and error response, that is, the performance outcome as adjusted by a number of other factors. In the present study, instantaneous steering angle indicates raw reaction, while maximum right-side/left-side lane departures indicate driving performance. The former is precipitated by sympathetic arousal, while the latter is the result of higher-order modulation over the initial steering responses.

We hereby studied a comprehensive set of stressors through a three-level causal decomposition (arousal → reaction → error) in long/uneventful versus short/eventful time scales, arriving at intriguing conclusions about driving behaviors. These conclusions contribute to open questions in neuroscience regarding conflict resolution^9^, while they stand to inform human-car interfaces, tying them to traffic regulations of the future.

## Results

We analyzed the association of perinasal perspiration **E** with steering angle **ST** and maximum departure on the right (*X*_*R*_) and left (*X*_*L*_) side of the lane based on results from a driving simulator experiment on *n* = 59 subjects. This reflected the association of sympathetic state with steering performance (indicative of motor reaction) and driving performance. The experiment had three parts:

Introductory Sessions: We measured perinasal perspiration during a sitting session with soothing music to establish the subject’s sympathetic baseline at rest. This was followed by two simulator drives, where we also measured perinasal perspiration. The first drive familiarized subjects with the simulator (Practice Drive, or PD). The second drive intended to relax subjects (Relaxing Drive, or RD) by having them operate the vehicle in a simple environment.

Loaded Drives: We measured perinasal perspiration, steering angle, and maximum right-side/left-side lane departures on four drives (order randomized) repeated on the same highway segment under similar ambient conditions; they featured a modicum of driving difficulty (Loaded Drives, or LD_*j*_, where *j* denotes the type of stressor). One loaded drive had no additional stressor (LD_∅_, *j* = ∅). Each of the remaining three loaded drives was characterized by a different stressor *j*: Loaded Drive with Cognitive Stressor (LD_*C*_, *j* = *C*); Loaded Drive with Emotional Stressor (LD_*E*_, *j* = *E*); Loaded Drive with Sensorimotor Stressor (LD_*M*_, *j* = *M*). The specific stressor *j* was applied twice during the corresponding drive LD_*j*_. The presence or absence of the specific stressor divided the LD_*j*_ drives into five phases: 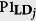 (no stressor for all); 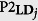 (no stressor in LD_∅_ | stressor in LD_*C*_, LD_*E*_, LD_*M*_); 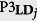 (no stressor for all); 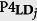 (no stressor in LD_∅_ | stressor in LD_*C*_, LD_*E*_, LD_*M*_); 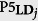 (no stressor for all). Table 1(a) illustrates the stressor layout for the Loaded Drives.

Failure Drive: We measured perinasal perspiration, steering angle, and maximum right-side/left-side lane departures on a drive that featured at the end an unintended acceleration incident, during which the vehicle brake had no effect (Failure Drive or FD_*y*_, where *y* denotes the arm of the experiment). Subjects belonging to the *y* = *o* arm of the experiment did not have any additional stressor during the FD_*y*_ drive. Subjects belonging to the *y* = *L* arm of the experiment had an additional stressor during the second half of the FD_*y*_ drive. This stressor was of mixed distracting nature - initially sensorimotor, and then mixed with cognitive alternating with emotional stimuli. The presence or absence of the mixed stressor and the occurrence of the dramatic failure divided the FD_*y*_ drive into three phases: 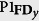 (no stressor for all); 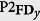 (no stressor for *y* = *o* vs. mixed stressor for *y* = *L*); 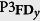 (failure event for all). Table 1(b) illustrates the stressor/event layout for the two experimental arms in the Failure Drive.

### Experimental Validity

Trait Anxiety Inventory (TAI)^10^ and Personality A/B^11^ scores for the subjects whose data were analyzed in this research (*n* = 59), covered a broad range in the non-extreme regions of the respective scales. Specifically, the TAI scores ranged from 20 to 52 in a scale graded from 20 to 80; mean TAI was 33.22 and the standard deviation was 7.87. The A/B scores ranged from 144 to 282 in a scale graded from 35 to 380; mean A/B was 208.85 and the standard deviation was 30.08.

We found no significant correlation of TAI and A/B scores with mean perinasal perspiration responses or mean absolute steering or maximum right-side/left-side lane departures in any of the drives (*p* > 0.05 for the correlation coefficients in all cases). This suggests that key personality traits that could have biased sympathetic responses, driver reactions, and driver performance did not play any role.

To ascertain that the experiment’s challenging drives were perceived as such, we asked subjects to complete the NASA Task Load Index (TLX) after each drive. NASA TLX measures subjective workload assessment on operators working with man-machine interfaces. It draws on six sub-scales TLX_*s*_: Mental Demand (TLX_*MD*_, *s* = *MD*), Physical Demand (TLX_*PD*_, *s* = *PD*), Temporal Demand (TLX_*TD*_, *s* = *TD*), Performance (TLX_*P*_, *s* = *P*), Effort (TLX_*E*_, *s* = *E*), and Frustration (TLX_*F*_, *s* = *F*).

We ran a mixed effects model to examine the dependence of each sub-scale TLX_*s*_ on fixed effects, defined by the different types of stressful loaded drives (LD_*C*_, LD_*E*_, LD_*M*_), keeping the loaded drive with no stressors LD_∅_ as the intercept:


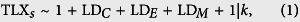


where *k* stands for subjects, acting as random effects. The model indicated that loaded drives LD_*C*_ and LD_*M*_ with cognitive and sensorimotor stressors, respectively, had significantly higher scores with respect to LD_∅_ in all NASA TLX sub-scales (*p* < 0.001 for all TLX_*s*_). The model also indicated that the emotionally loaded drive LD_*E*_ had significantly higher scores with respect to LD_∅_ in the Mental Demand, Temporal Demand, and Effort sub-scales only (*p* < 0.01 for TLX_*MD*_, TLX_*TD*_, TLX_*E*_).

These results suggest that subjects perceived drives with cognitive or sensorimotor loads as challenging across the sub-scales of a validated instrument^12^, thus, confirming the effectiveness of the study’s design regarding these two stressors. The fact that subjects perceived the emotionally loaded drives as challenging in half of the NASA’s instrument sub-scales, suggests that the study’s design was moderately effective in this respect.

### Introductory Sessions Analysis

Comparison among the mean perinasal perspiration signals in the baseline session, the practice drive, and the relaxing drive indicated the absence of any significant differences (*p* > 0.05, repeated measures analysis of variance). This suggests that in all these cases subjects were hovering close to their tonic levels, in the absence of any serious challenge.

### Loaded Drives Analysis

We analyzed perinasal perspiration (explanatory variable) to determine the sympathetic effect of distractions in loaded drives. Next, we analyzed steering angle (response variable I) to determine how sympathetic effects associate with motor reactions. We also tested and found that age significantly affected the absolute mean steering angle, with older subjects exhibiting larger values (*p* < 0.05). Finally, we analyzed maximum right-side/left-side lane departures (response variables II) to ascertain how motor reactions are modulated, shaping error prone driving behaviors.

Specifically, for the explanatory variable we computed the mean perinasal perspiration signal intensity 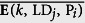 for each driving phase P_*i*_, of each loaded drive LD_*j*_, for each subject *k*. These values represented the mean sympathetic arousals exhibited by the subjects in response to the presence or absence of stress stimuli.

For response variable I, we computed the mean angular steering deviation (in absolute terms) 
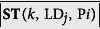
 for each driving phase P_*i*_, of each loaded drive LD_*j*_, for each subject *k*. These steering values served as indicators of motor reactions. Given that the subjects were traveling on a straight highway for 10.4 out of the 10.9 km of the drive, the mean absolute steering value should have been close to zero; the further away from zero, the stronger the sympathetic effect on instantaneous motor responses - an apparent deterioration of steering performance.

For response variables II, we computed the maximum lane departures on the right *X*_*R*_(*k*, LD_*j*_, P_*i*_) and left *X*_*L*_(*k*, LD_*j*_, P_*i*_) side of the road for each driving phase P_*i*_, of each loaded drive LD_*j*_, for each subject *k*. Here we define lane departure as the position of the car’s center with respect to the right or left boundary of the road, depending on the side it veered off. Ideally, the driver should maintain a nearly constant distance from these boundaries, driving in the middle of her/his lane (*X*_*R*_ ≈ *X*_*L*_ ≈ 0). If her/his car’s lateral position deviates significantly, then the maximum lane departure values *X*_*R*_ and/or *X*_*L*_ would increase substantially - a true deterioration of driving performance.

For each subject, we normalized the explanatory and response variables with respect to the corresponding variables in the LD_∅_ drive that featured no stressor. These LD_∅_ baselines represented the subject’s sympathetic state, steering performance, and driving performance under normal conditions. Since the itinerary and environment remained the same in all loaded drives, any mean deviations from the subject’s LD_∅_ baselines should be attributed to the forced distractions.

### Effect of Cognitive Load on Sympathetic State, Steering and Driving Performance

For our sample of *n* = 59 subjects, we computed for each driving phase P_*i*_ the distributions of paired differences between:

• Mean perinasal perspiration in LD_*C*_ and LD_∅_ (Eq. 2) - proxy for sympathetic changes


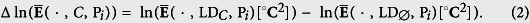


• Mean absolute steering angle in LD_*C*_ and LD_∅_ (Eq. 3) - proxy for steering changes


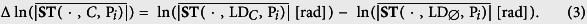


• Maximum lane departures in LD_*C*_ and LD_∅_ on the right and left side (Eq. 4a–4b, 15) - proxies for driving changes


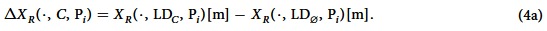



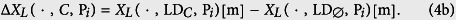


**Figure 1:**
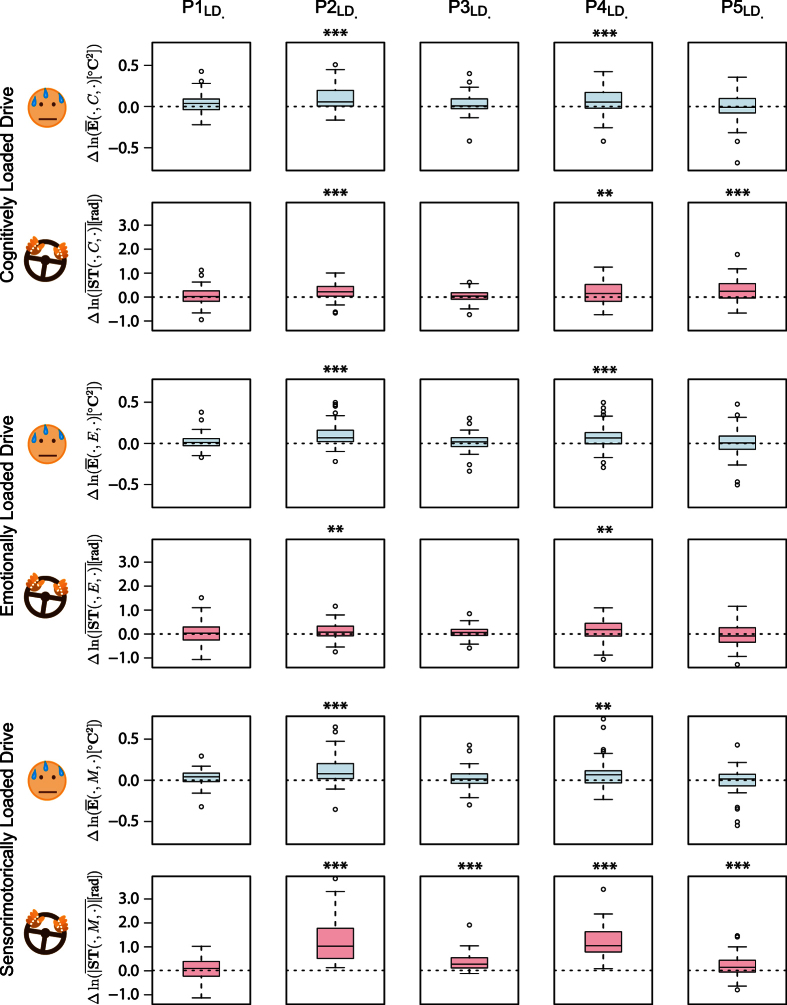
Paired t-tests for the explanatory (perinasal perspiration) and response I (steering) variables in each phase of the cognitively, emotionally, and sensorimotorically loaded drives.

Equation (2) produced the first row of boxplots in Fig. 1, suggesting that cognitive distraction of subjects in phases 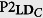 and 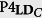 had as a result significant elevation of their mean sympathetic arousal, with respect to phases 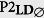 and 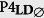 in the no-stressor drive (*p* < 0.001, paired t-tests in both cases).

Equation (3) produced the second row of boxplots in Fig. 1, suggesting that cognitive distraction of subjects in phases 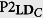 and 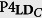 had as a result significant deterioration in mean steering performance, always with respect to phases 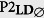 and 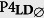 in the no-stressor drive (*p* < 0.001 in 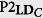 and *p* < 0.0125 in 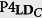, paired t-tests in both cases). It is interesting that deterioration in mean steering performance remained significant in phase 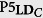 with respect to phase 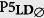 in the no-stressor drive (*p* < 0.001, paired t-test), indicating that there was a lingering behavioral effect on subjects with respect to response variable I, which outlived the second application of the cognitive stressor.

**Figure 2:**
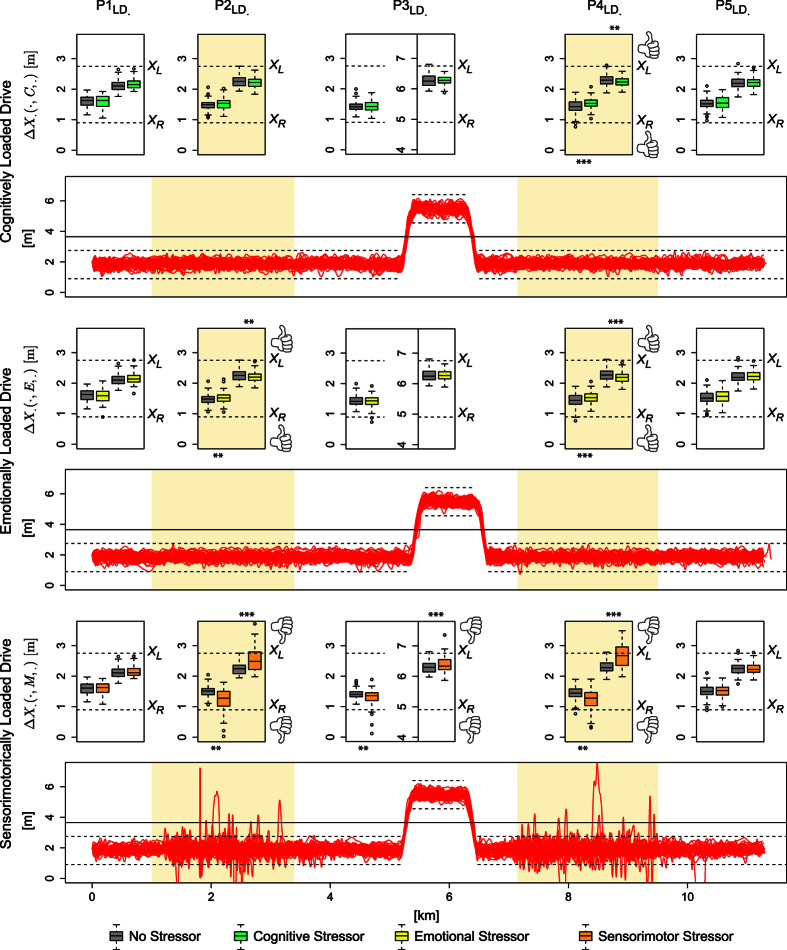
Paired t-tests for the response II variables (maximum R/L lane departures) in each phase of the cognitively, emotionally, and sensorimotorically loaded drives wrt the no-stressor drive; they are accompanied by visualizations of the cars’ itineraries in each stressful drive. Where color boxplots are closer to the R/L boundaries wrt grey boxplots, the stressor had a negative effect.

Equations (4a–4b), 15 produced the first row of boxplots in Fig. 2, suggesting that cognitive distraction of subjects in phase 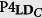 had as a result significant improvement in terms of maximum right-side and left-side lane departures, with respect to phase 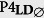 in the no-stressor drive (*p* < 0.001 for *X*_*R*_ in 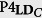 and *p* < 0.0125 for *X*_*L*_ in 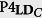, paired t-tests in both cases).

### Effect of Emotional Load on Sympathetic State, Steering and Driving Performance

For our sample of *n* = 59 subjects, we computed for each driving phase P_*i*_ the distributions of paired differences between:

• Mean perinasal perspiration in LD_*E*_ and LD_∅_ (Eq. 5) - proxy for sympathetic changes


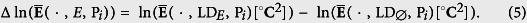


• Mean absolute steering angle in LD_*E*_ and LD_∅_ (Eq. 6) - proxy for steering changes


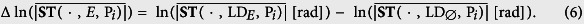


• Maximum lane departures in LD_*E*_ and LD_∅_ on the right and left side (Eq. 7a–7b, 35) - proxies for driving changes


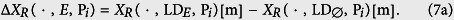



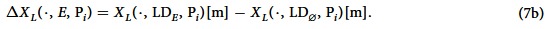


Equation (5) produced the third row of boxplots in Fig. 1, suggesting that emotional distraction of subjects in phases 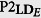 and 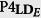 had as a result significant elevation of their mean sympathetic arousal, with respect to phases 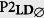 and 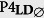 in the no-stressor drive (*p* < 0.001, paired t-tests in both cases).

Equation (6) produced the fourth row of boxplots in Fig. 1, suggesting that emotional distraction of subjects in phases 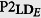 and 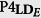 had as a result significant deterioration in mean steering performance, always with respect to phases 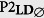 and 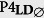 in the no-stressor drive (*p* < 0.0125, paired t-tests in both cases).

Equations (7a–7b), 35 produced the second row of boxplots in Fig. 2, suggesting that emotional distraction of subjects in phases 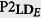 and 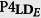 had as a result significant improvement in terms of maximum right-side and left-side lane departures, with respect to phases 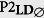 and 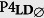 in the no-stressor drive (*p* < 0.0125 for *X*_*R*_ and *X*_*L*_ in 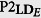 and *p* < 0.001 for *X*_*R*_ and *X*_*L*_ in 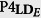, paired t-tests in all cases).

### Effect of Sensorimotor Load on Sympathetic State, Steering and Driving Performance

For our sample of *n* = 59 subjects, we computed for each driving phase P_*i*_ the distributions of paired differences between:

• Mean perinasal perspiration in LD_*M*_ and LD_∅_ (Eq. 8) - proxy for sympathetic changes


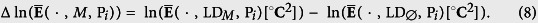


• Mean absolute steering angle in LD_*M*_ and LD_∅_ (Eq. 9) - proxy for steering changes


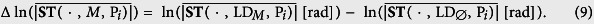


• Maximum lane departures in LD_*M*_ and LD_∅_ on the right and left side (Eq. 10a–10b, 53) - proxies for driving changes


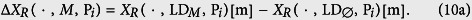



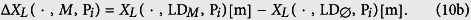


Equation (8) produced the fifth row of boxplots in Fig. 1, suggesting that sensorimotor distraction of subjects in phases 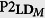 and 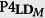 had as a result significant elevation of their mean sympathetic arousal, with respect to phases 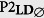 and 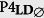 in the no-stressor drive (*p* < 0.001 in 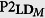 and *p* < 0.0125 in 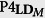, paired t-tests in both cases).

Equation (9) produced the sixth row of boxplots in Fig. 1, suggesting that sensorimotor distraction of subjects in phases 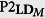 and 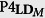 had as a result significant deterioration in mean steering performance, always with respect to phases 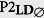 and 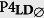 in the no-stressor drive (*p* < 0.001, paired t-tests in both cases). It is interesting that deterioration in mean steering performance remained significant in phases 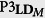 and 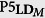 with respect to phases 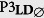 and 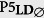 in the no-stressor drive (*p* < 0.001, paired t-tests in both cases), indicating that there was a lingering behavioral effect on subjects with respect to response variable I, which outlived each application of the sensorimotor stressor.

Equations (10a–10b), 53 produced the third row of boxplots in Fig. 2, suggesting that sensorimotor distraction of subjects in phases 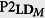 and 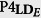 had as a result significant deterioration in terms of right and left lane departures, with respect to phases 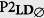 and 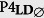 in the no-stressor drive (*p* < 0.0125 for *X*_*R*_ in 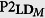 and 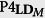, *p* < 0.001 for *X*_*L*_ in 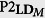 and 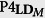, paired t-tests in all cases). This behavioral effect on subjects with respect to response variables *X*_*R*_ and *X*_*L*_ tended to linger in 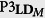, outliving the first application of the sensorimotor stressor (*p* < 0.0125 for *X*_*R*_ and *p* < 0.001 for *X*_*L*_ in 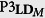, paired t-tests in both cases).

### Failure Drive Analysis

**Figure 3:**
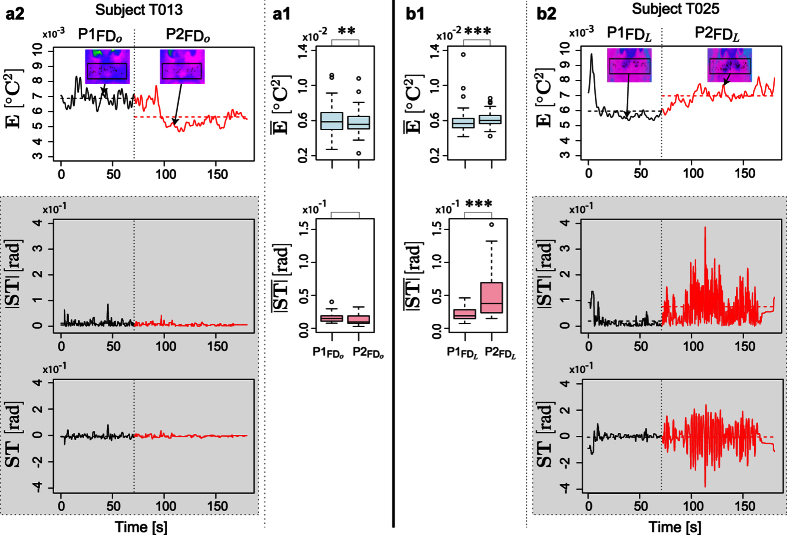
Statistics for Failure Drive’s first two phases. Mean perinasal perspiration and absolute steering distributions for: (**a1**) The *y* = *o* group. (**b1**) The *y* = *L* group. § Failure Drive examples. Perinasal perspiration (***E***) and absolute steering/steering (|**ST**|/**ST**) signals for: (**a2**) Subject T013 belonging to the *y* = *o* group. The two insets depict thermal snapshots of perinasal perspiration in the initial (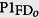) and later (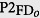) phase of the drive; the first pattern appears busier than the second. (**b2**) Subject T025 belonging to the *y* = *L* group. The two insets depict thermal snapshots of perinasal perspiration in the initial (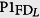) and later (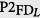) phase of the drive; the second pattern appears busier than the first. The dashed horizontal lines indicate the mean values in the corresponding signal segments.

In the Failure Drive, for the *y* = *o* group we found that phase 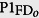 featured significantly higher values than phase 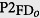 in terms of mean sympathetic responses (*p* < 0.0125, paired t-test - Fig. 3a1). In contradistinction, for the *y* = *L* group we found that phase 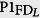 featured significantly lower values than phase 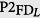 both in terms of mean sympathetic and mean absolute steering signals (*p* < 0.001, paired t-tests in both cases - Fig. 3b1).

Figure 3a2,b2 depict sympathetic (i.e., perinasal perspiration) and absolute steering signals from subjects representative of the *y* = *o* and *y* = *L* groups, respectively. In the *y* = *o* example, the subject’s sympathetic signal was somewhat elevated in phase 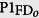 with respect to phase 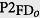 in the mean sense. This could be ascribed to the start-up effect, where the sympathetic system responds to the driving transition from idle to a steady state. In the *y* = *L* example, however, the sympathetic and absolute steering signals during phase 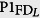 (non-loaded phase) were significantly lower with respect to phase 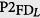 (loaded phase) in the mean sense. Specifically, during the loaded portion of the drive the steering signal became highly variable, indicating that the driver had frequently strong impulsive motor responses. Apparently, the dramatic effect of the mixed stressor during the loaded phase 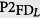 of the Failure Drive overwhelmed any start-up effect characterizing its initial non-loaded phase 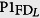.

Running a mixed effects model to examine the dependence of mean absolute steering 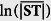 on fixed effects:


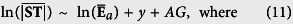


**Figure 4:**
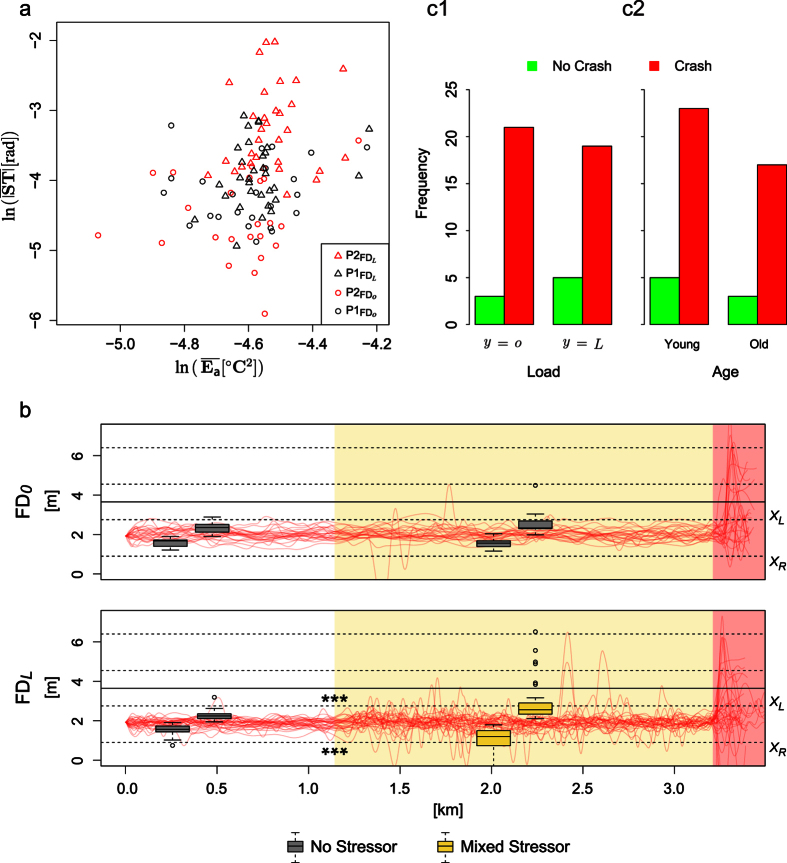
(**a**) Scatterplot of mean absolute steering vs. mean perinasal perspiration for all subjects in the first two phases of FD. (**b**) Car itineraries for the *y* = *o* and *y* = *L* groups. White, yellow, and pink pavement indicate phase 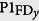, 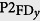, and 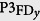, respectively. Lane departures in 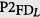, where the mixed stressor applied, are striking, suggesting an increased risk of accident. Extreme lane departures in 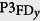 are the results of the startling incidence - almost all cars crashed. Crash - No Crash incidents following the unintended acceleration event in phase 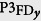 according to: (**c1**) Load. (**c2**) Age.

• 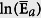: mean perinasal perspiration adjusted to each subject’s resting baseline,

• *y*: sympathetic load indicator (*y* = *o* vs. *y* = *L*),

• *AG*: age group (Young: <27 vs. Old: >60),

we found that all three variables representing fixed effects bore significance in predicting steering behavior (*p* < 0.05); subjects were treated as random effects. Furthermore, the coefficients in all cases were positive. Hence, for each of the load and age binary variables as we moved from the low level (*y* = *o* and *AG* = Y*o*ung) to the high level (*y* = *L* and *AG* = Old), steering increased. Importantly, for fixed *y* and *AG*, higher values of mean perinasal perspiration (indicating elevated arousal) were correlated with higher mean absolute steering values (Fig. 4a). Figure 4b shows how higher mean absolute steering values under the spell of the mixed stressor in 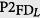, resulted in ominous lane departures and outright traffic violations; the difference from the orderly itinerary patterns in 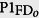, 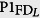, and 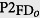 is striking.

Specifically, mixed distraction of subjects in phase 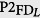 had as a result highly significant deterioration in terms of maximum right-side and left-side lane departures, with respect to phase 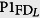 where no-stressor applied (*p* < 0.001 for *X*_*R*_ and *X*_*L*_ in both cases). By contrast, there was no significant deterioration in terms of maximum right-side and left-side lane departures in phase 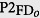 with respect to 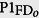, for the *y* = *o* (no stressor) arm of the experiment.

It is important to note that we adjusted the individual perinasal perspiration signals by subtracting the mean tonic level (resting baseline) of each subject, correspondingly. This was necessary for fair comparison, as each person had a different sympathetic baseline and what was of interest was to find how much the drive and the distractions raised the subject’s sympathetic state above her/his resting baseline.

Regarding the unintended acceleration event that took place the last 10 s of the Failure Drive (phase 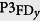), the question was if prior distraction or any other factor, played a role in the subject’s crash risk *CR*. Running a logistic regression model to examine the dependence of crash risk *CR* on fixed effects:


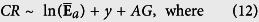


• 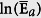: mean perinasal perspiration adjusted to each subject’s resting baseline,

• *y*: sympathetic load indicator (*y* = *o* vs. *y* = *L*),

• *AG*: age group (Young: <27 vs. Old: >60),

we found that all variables representing fixed effects had no significance in predicting crash odds (*p* > 0.05). In fact, as Fig. 4c1,c2 illustrate, a very small number of drivers avoided crashing, among those who experienced the unintended acceleration event.

## Discussion

Driver safety is ensured when the driver operates the vehicle sensibly and her/his environment does not change abruptly. An interesting question is what happens if either of these conditions is not met. Here we restricted ourselves to the study of distracted driving and unintended acceleration. Although distracted driving is not the only form of non-sensible driving (e.g., driving under intoxication is another infamous variety), it is certainly the most prevalent, especially during rush hours, when the individual effects on traffic flow are maximized. By the same token, unintended acceleration is not the only form of dramatic startle. However, because it can have a devastating impact in busy thoroughfares where the room for error is small, it delineates the envelope of disastrous events requiring responses at the level of neurophysiological latency.

Pivotal to our approach is the abstraction of distracted driving into three main categories depending on the stressor involved, that is, cognitive, emotional, and sensorimotor. This is a comprehensive and highly diverse stressor set; thus, sensing its physiological effects through a universal indicator can streamline the measurement process, rendering future applications practical. We used perinasal perspiration to measure sympathetic arousal - a prime physiological response to stress, independent of the stressor type. We extracted the perspiratory signal using a clinically validated method based on thermal imaging^8^. This sensing modality rendered the physiological measurement process totally unobtrusive.

Furthermore, we measured the direct sympathetic effect on motor reactivity using the instantaneous absolute value of the steering angle. We also measured the filtered effect on driving using the maximum lane departures on the right and left side of the road - an indicator that tracks propensity for error and thus, risk for accident.

In a simulator experiment, designed to isolate each stressor type, we found that all three stressors resulted in significant increases of the drivers’ sympathetic arousal levels, all other things being equal (i.e., itinerary and traffic conditions). Furthermore, we found that these elevated arousal levels were associated with significant increases in the mean value of the absolute steering signal - an indication of erratic reaction directly driven by sympathetic arousal. Interestingly, we also found that these erratic reactions were fully corrected when the hand-eye feedback loop was not interrupted; this was true in the cognitive and emotional stressor cases. For example, in the case depicted at the bottom right panel of Fig. 3, where a mixed stressor was at work, the steering signal is mostly symmetric, with a notable asymmetry in the middle, when the subject was engaged in texting. A likely explanation for this paradox is that cognitive or emotional conflict activated the anterior cingulate cortex (ACC), which successfully counter-balanced erroneous motor reactions. In the case of pure or mixed sensorimotor conflict, however, where the hand-eye feedback loop was interrupted, ACC filtering slipped, failing at times to counterbalance instinctive motor reactions and thus, resulting into occasional lane departures.

Hence, moderate levels of pure cognitive or emotional loading have either beneficial effects or at worst no effects on apparent vehicle control, although they continue to affect instinctive (and hidden) driver responses - i.e., instantaneous steering. Although this result is intriguing, one should keep in mind that almost certainly extreme cognitive and emotional loads would tilt the scale towards unsafe driving behaviors. The question is where is the threshold, a problem that would be difficult to solve in the lab due to ethical considerations. We also acknowledge that despite randomization, there may have been a residual practice effect in the four loaded drives of the experiment, which perplexes things further. The answer may come from massive anonymous observations in actual cars, when these methods are applied at large scale.

Furthermore, this first simulation experiment shed light on the likely neurophysiological mechanism that renders sensorimotor stressors, such as texting while driving, so disruptive and dangerous, even in moderate amounts; they knock out human’s last line of conflict resolution defense, that is, the anterior cingulate cortex.

In this respect, one should note that although driving under moderate cognitive or emotional loads is technically optimal, it carries hidden risks. Indeed, as the second simulation experiment demonstrated, when these loads mix with sensorimotor distractions result into massive errors, releasing unchecked all the pressure kept under the lid by ACC.

The second simulation experiment also gave us the opportunity to investigate driving performance in unintended acceleration incidents amidst busy thoroughfares. In such cases, we found that the absence or presence of stressors and the accompanying physiological responses, as well as age, played no role whatsoever in the crash outcome. This suggests that the compensatory action of ACC operates at a time scale above neurophysiological latency and thus, is unable to render an instant hand in startling events. A future promising direction of research would be to test if enhancing driver training with startle handling would have any effect on crash odds. Current driver training is lacking in this respect.

A limitation of the perinasal imaging method is that it does not perform reliably when the subject has facial hair. For this reason about 13% of the original data set (nine male subjects) could not be processed. Other methods for peripheral sensing of sympathetic arousal, such as palmar electrodermal activity sensing, have their own set of problems, especially in the context of driving where the subjects’ hands are engaged. Further research into measurement methods will solve these problems in due course. What the current study convincingly demonstrates, however, is that sensorimotor distractions over-arouse the average driver and may result in significant deterioration of her/his driving performance. Furthermore, real-time unobtrusive measurement of driver’s arousal and its behavioral effects are within reach, opening the way for engineering orthotic feedback loops. These loops will notify drivers (and perhaps others in the vicinity) of their predicament.

## Methods

### Ethics Statement

The experimental procedures were approved by the Institutional Review Boards (IRB) of the University of Houston and the Texas A&M University. The authors performed these procedures in accordance with the approved guidelines, obtaining informed consent from each subject before conducting the experiments.

### Subjects

We recruited subjects from the Bryan and College Station, TX communities (population about 250,000) through email solicitations and flyer postings. Subjects had a valid driving license and had normal or corrected to normal vision. We restricted admission to individuals with at least one and a half years of driving experience who were between 18 and 27 years of age (Young cohort) or above 60 years of age (Old cohort). We excluded subjects on medications affecting their ability to drive safely. A total of *n* = 78 subjects conforming to the inclusion-exclusion criteria volunteered for the study. One subject quit in the middle of the experiment because of motion sickness; and, data for *n* = 9 subjects were not recorded properly due to technical issues. Hence, data for only *n* = 68 subjects (35 male/33 female) were complete and problem free. We performed analysis on *n* = 59 (26 male/33 female) of these subjects. We could not perform analysis on *n* = 9 male subjects because they had facial hair, rendering extraction of the perinasal perspiration signal problematic. The age composition of the analyzed data set was balanced, with 12 males/18 females belonging to the Young cohort and 14 males/15 females belonging to the Old cohort.

### Experimental Design

In a high fidelity driving simulator manufactured by Realtime Technologies, Inc (Supplement Fig. 1), we ran a controlled experiment randomly assigning subjects to two groups: Nonloaded group (*y* = *o*, *n* = 26) and Loaded group (*y* = *L*, *n* = 33). This group categorization related to the two arms of the last session in the experiment. All other experimental sessions were the same for both groups. Upon signing the consent form, the subjects completed three questionnaires:

#### Biographic Questionnarie

It identified key facts about the subject, such as gender, age, and driving record.

#### Trait Anxiety Inventory

Long-standing stress might have an effect on sympathetic responses and thus, scoring trend anxiety was of potential interest to this study^10^.

#### Personality Type A/B

This was a modified version of the Jenkins Activity Survey^11^. Some studies have shown association between type A personalities and specific driving behaviors^13^; thus, scoring of type A/B personalities was also of potential interest to this study.

Next, the subjects went through *T*_*session*_ = 8 experimental sessions. The first one was a baseline session (1: BS) where the subjects sat quietly in a dimly lit room, listening to soothing music for 5 min. Following this baseline session, the subjects went through seven driving sessions on the simulator. In order of execution, the drives were as follows:

2: Practice Drive (PD): The subjects familiarized themselves with the simulator by driving on a 8 km straight section of a four-lane highway at posted speeds; two lanes were dedicated to traffic in each direction, with the subject’s car traveling in the right lane (R); the speed limits changed every couple of kilometers (80 kph → 50 kph → 100 kph) - Supplement Fig. S2.

3: Relaxing Drive (RD): The subjects had to drive on a 10.9 km straight section of a four-lane highway with posted speed limit of 70 kph; two lanes were dedicated to traffic in each direction, with the subject’s car traveling in the right lane (R); there was light traffic on the oncoming lanes (~3 vehicles per km). The subjects were forced to change lane (R to L) after 5.2 km into the drive. They stayed in the left lane (L) for 1.2 km, before they were directed back to the right lane (R) - Supplement Fig. S3. The rationale for this lane change was to reduce the monotony of the drive.

4–7: Loaded Drives: We randomized the order of four special driving sessions, called ‘loaded’ drives, featuring the same challenging driving conditions (construction zones - Supplement Fig. S4). Each loaded drive was uniquely characterized by an additional stressor or absence thereof. This stressor assumed the form of a secondary activity that was forced in two phases during the course of the drive. All loaded drives were on the same 10.9 km section of a four-lane highway with posted speed limit of 70 kph; two lanes were dedicated to traffic in each direction, with the subject’s car traveling in the right lane (R). The drives featured heavy traffic on the oncoming lanes (>12 vehicles per km), construction on the left lane (L), and traffic delineator posts on both sides of the right lane (R). The subjects were forced to change lane (R to L) after 4.4 km into the drive. They stayed in the left lane (L) for 1.2 km, before they were directed back to the right lane (R). During the detour, construction cones appeared on the right side of the lane. In more detail, the loaded drives were as follows:

- **Loaded Drive:** (LD_∅_) Driving with no secondary activity (no additional stressor).
- **Cognitive Drive:** (LD_*C*_) Driving under a cognitive stressor. The cognitive stressor was mathematical questions (Supplement: Cognitive Stressor - Mathematical Questions) in one phase of the drive and analytical questions (Supplement: Cognitive Stressor - Analytical Questions) in another phase of the drive, posed orally by the experimenter. The experimenter started posing these questions from the beginning of the relevant list, stopping only when the phase time was over. The subjects had to answer the questions to the best of their abilities. The mathematical vs. analytical phase order was randomized.
- **Emotional Drive:** (LD_*E*_) Driving under an emotional stressor. The emotional stressor was emotionally stirring questions posed orally by the experimenter in two phases. There were two sets of questions: a set with less pointed questions (Supplement: Emotional Stressor - Basic Questions) and a set with more pointed questions (Supplement: Emotional Stressor - Pointed Questions). In the first phase the experimenter was asking basic questions for 20 s, starting from the beginning of the relevant list. The remaining time s/he was asking pointed questions, starting from the beginning of the relevant list. In the second phase the experimenter continued for 30 s with basic questions, starting form the point s/he left in Phase 1. The remaining time the experimenter was asking pointed questions, starting from the point s/he left in Phase 1. The subjects had to answer all these questions to the best of their abilities.
- **Sensorimotor Drive:** (LD_*M*_) Driving under a sensorimotor stressor. The sensorimotor stressor was texting back words, sent one by one to the subject’s smartphone; this texting exchange took place in two phases.

The phase layout within each stressful LD_*j*_ drive (*j* ∈ [*C*, *E*, *M*]) was as follows:

- **Phase** 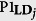: Driving without distractions for ~80 s.
- **Phase** 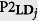: Driving while engaging in a secondary activity *j* for ~160 s.
- **Phase** 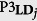: Driving without distractions for ~240 s (coincided with the detour).
- **Phase** 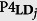: Driving while engaging in a secondary activity *j* for ~160 s.
- **Phase** 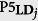: Driving without distractions for ~120 s.

8: Failure Drive (FD): Subjects had to drive a 3.2 km highway section identical to the last 3.2 km segment of the loaded drives. Subjects belonging to the *y* = *o* group did not engage in any secondary activity (Supplement Fig. S5). Subjects belonging to the *y* = *L* group, however, drove under mixed stressors the last 2 km of the drive (Supplement Fig. S6). Initially, *y* = *L* subjects had to text back a sentence that appeared in their smartphone; then, they had to respond to an alternating series of mathematical/analytical and emotional questions posed orally by the experimenter while they kept texting. Towards the end of the drive, all subjects had to wait on a red light at an intersection. Prior to the green signal a vehicle malfunction resulted into an unintended acceleration incident, propelling the car forward and putting it on a collision course with another car that had entered the intersection. The subject had 5 s to react before a collision. Hence, the FD drive had three phases 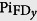 (*i* ∈ [1, 2, 3], *y* ∈ [*o*, *L*]):

- **Phase** 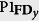: First half of drive - no distractions.
- **Phase** 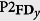: Second half of drive - *y* = *o* no distractions; *y* = *L* mixed distractions.
- **Phase** 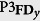: Experiencing an unintended acceleration incident for ~11 s.

There was a 2 min break between drives. During each break, subjects were completing the NASA Task Load Index (TLX) for the preceding drive. NASA-TLX is a subjective workload assessment tool that complements the objective assessment of task-induced sympathetic arousal, captured via thermal imaging. NASA-TLX features a multi-dimensional rating procedure that derives an overall workload score based on a weighted average of ratings on six sub-scales. These sub-scales include Mental Demands, Physical Demands, Temporal Demands, Own Performance, Effort, and Frustration^12^.

During the baseline session and all the subsequent drives, we continuously imaged the subject’s face with a thermal camera (Supplement Fig. S1). At the same time, we programmed the simulator to save a record of the evolving driving parameters. These parameters included speed, acceleration, braking, steering angle, and lane position.

We used the Tau 640 thermal camera (FLIR Commercial Systems, Goleta, CA); it features a small size (44 × 44 × 30 mm), a reasonable price (<$5,000), and adequate thermal (<50 mK) and spatial resolution (640 × 512 pixels). Based on the experimental protocol, the total number of thermal clips *C*_*thermal*_ should have been: *C*_*thermal*_ = *n* × *T*_*session*_ = 59 × 8 = 472. However, only *C*_*thermal*_ = 469 clips have been collected and used in the statistical analysis. The missing clips were corrupted due to technical problems. The missing data is a tiny portion of the total data set and within the range of expected loss in a realistic study. Given their random distribution, they do not affect the statistical validity of the results.

Algorithmic processing of the thermal imagery yielded a signal that quantified perinasal perspiration. The algorithm included a virtual tissue tracker that kept track of the region of interest, despite the subject’s small motions. This ensured that the physiological signal extractor operated on consistent and valid sets of data over the clip’s timeline.

### Statistical Analysis

We applied statistics using the freeware program R, version 3.1.2 (http://www.r-project.org). We measured the strength of the linear relationships on the scatterplots with the Pearson’s product moment correlation coefficient and performed the respective test of significance at *α* = 0.05. We did hypothesis testing against a two-tail alternative, setting levels of significance at *α* = 0.0125 designated by **, or *α* = 0.001 designated by ***. The *α* = 0.0125 is Bonferroni-corrected for *n* = 4 comparisons, referring to the four variables we used to characterize drivers: perinasal perspiration, absolute steering angle, maximum lane departure on the right, and maximum lane departure on the left.

### Thermal Imaging - Tissue Tracking

We used the tissue tracker reported in Zhou *et al.*^14^. On the initial frame, the user initiates the tracking algorithm by selecting the upper orbicularis oris portion of the perinasal region. The tracker estimates the best matching block in every next frame of the thermal clip via spatio-temporal smoothing (Supplement Fig. S7). A morphology-based algorithm is applied on the evolving region of interest to compute the perspiration signal. Any high-frequency noise in this signal is suppressed by a Fast Fourier Transformation (FFT) filter.

### Thermal Imaging - Perinasal Signal Extraction

A key method of this study is the extraction of the perinasal perspiration signal from the thermal imagery; this is the sympathetic indicator used. Supplement-Fig. S7 shows the thermal signature of perspiration spots on the perinasal area of a subject in moments of low and high excitation. In facial thermal imagery, activated perspiration pores appear as small ‘cold’ (dark) spots, amidst substantial background clutter. The latter is the thermo-physiological manifestation of the metabolic processes in the surrounding tissue. We quantified this spatial frequency pattern by extracting an energy signal **E**(*k*, *j*, *i*), indicative of perspiration activity in the perinasal area of subject *k*, for session *j*, and phase *i*. We computed this signal by applying the clinically validated morphological method reported by Shastri *et al.*^8^.

## Additional Information

**How to cite this article**: Pavlidis, I. *et al.* Dissecting Driver Behaviors Under Cognitive, Emotional, Sensorimotor, and Mixed Stressors. *Sci. Rep.* **6**, 25651; doi: 10.1038/srep25651 (2016).

## Supplementary Material

Supplementary Information

## Figures and Tables

**Table 1:**
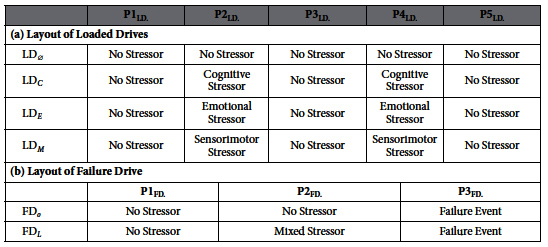
Experimental Design. Experimental sessions are crossovered in (a) and parallel grouped in (b).
