# Supplementary material for: Dissecting Driver Behaviors Under Cognitive, Emotional, Sensorimotor, and Mixed Stressors
Source: Sci Rep. 2016 May 12;6:25651. doi: 10.1038/srep25651 (PMC4867684; doi:10.1038/srep25651)
Supplement: Supplementary Information [file srep25651-s1.pdf]

Dissecting Driver Behaviors Under Cognitive, Emotional, Sensorimotor, and Mixed Stressors

I. Pavlidis, M. Dcosta, S. Taamneh, M. Manser, T. Ferris, R. Wunderlich, E. Akleman, P. Tsiamyrtzis

Supplementary Material

Visualization of the study’s entire data set can be accessed at:

http://subjectbook.times.uh.edu/displaySubject?studyNo=11&SubjectId=

Questionnaires used as Distracting Stressors

Cognitive Stressor - Mathematical Questions

[Ask questions until you see the relevant marker in the simulated world. Record with a check mark if answered correctly (correct answers are in parentheses). Record the incorrect answer if answered incorrectly.]

1. What is the sum of $34 + 78$? (112)
2. What is 2×36? (72)
3. What is $57 - 23$? (34)
4. What is 4×70? (280)
5. What is the sum of $54 + 23$? (77)
6. What is the sum of $48 + 31$? (79)
7. What is $91 - 33$? (58)
8. What is 85×6? (510)
9. What is 23×8? (184)
10. What is $67 - 23$? (44)
11. What is $365 \div 5$? (73)
12. What is the sum of $45 + 21$? (66)

13. What is $80 - 27$? (53)

14. What is $72 \div 12$? (6)

15. What is 14×8? (112)

16. What is the sum of $43 + 87$? (130)

17. What is 4×27? (108)

18. What is $75 - 32$? (43)

19. What is 6×17? (102)

20. What is the sum of $57 + 32$? (89)

21. What is the sum of $84 + 31$? (115)

22. What is $93 - 37$? (56)

23. What is 58×5? (290)

24. What is 34×4? (136)

25. What is $76 - 43$? (33)

Cognitive Stressor - Analytical Questions

[Ask questions until you see the relevant marker in the simulated world. Record with a check mark if answered correctly. Briefly write incorrect answer if answered incorrectly.]

1. How do you spell the word ‘simultaneously’?
2. Please describe two differences between College Station and Houston.
3. Why is Texas hotter than California?
4. Please describe three differences between a cat and a dog.
5. What is the difference between a sedan and an SUV?
6. Why is sea water salty?
7. Why can a bird fly?
8. Which state is the fourth largest state in the U.S.?
9. What are the colors on the flag of the United States?
10. How do you increase your running distance?
11. Which U.S. state is closest to Cuba?
12. What happens if you have a lack of exercise?
13. What is the difference between a sandwich and a burger?

14. My grandfather’s daughter hit her daughter. How do the daughters relate to each other?

15. How do you spell the word ‘accommodations’?

16. Please describe two differences between day and night.

17. Which U.S. state is the smallest?

18. Why do trees have leaves?

19. What is the difference between a mammal and a reptile?

20. How do you make a hot dog?

Emotional Stressor - Basic Questions

[Ask questions until 30 seconds have elapsed. Record a checkmark so you notate that you have already asked that question.]

1. Where were you born?
2. Are you male or female?
3. What is your nationality?
4. Did you have lunch today?
5. What is your first name?
6. What year were you born?
7. Can you speak Mandarin?
8. Do you have TV at home?
9. Do you have children?
10. Have you been to Asia before?
11. Do you like cats?
12. What is your hair color?
13. Do you like burgers?

14. Do you have a bank account?

15. Where do you live now?

16. How many cars do you own?

17. What is your eye color?

18. Did you go to the movies this week?

19. What is your mother’s first name?

20. Do you have siblings?

21. How old are you?

22. Are you good at playing guitar?

23. Have you eaten tacos before?

24. Did you watch the NBA this year?

25. Do you have a bachelor’s degree?

26. How tall are you?

27. Have you been to Colorado?

28. What kind of car do you drive?

29. Do you like to play video games?

30. Do you like sports?
31. What is your favorite movie?
32. What is your favorite color?
33. Do you like the outdoors?
34. Do you live in Texas?
35. What country are you in?
36. What is the color of grass?
37. Do you like music?
38. Do you have pets?

Emotional Stressor - Pointed Questions

[You have already asked 30 seconds of Basic questions. Ask questions until you see the relevant marker in the simulated world. Record a checkmark so you notate that you have already asked that question.]

1. What makes you a good friend to others?
2. How does seeing a baby cry make you feel?

3. Give an example of a time when you were angry with someone in the past and realized it was not their fault?
4. How shy or bold are you in a group setting?
5. What is a little white lie you have told to someone?
6. What are your educational or career goals in life?
7. What is the longest you've been in a relationship?
8. Give an example of when someone disagreed with you and you reacted in a way you now regret.
9. How much of a people pleaser are you?
10. Give an example of a recent deadline that you failed to meet.
11. Tell me about a positive memory with your family.
12. Do you prefer being the center of attention or would you prefer to be more in the background?
13. Give an example of when you have manipulated someone else to get what you want.
14. When was the last time you were late for an important appointment?
15. What are things (is something) that make(s) you really sad?
16. Who is someone who has trusted you with their secret?

17. Give an example of when you have taken credit for someone else's accomplishment.

18. Tell about a time you cheated on an exam or assignment, if ever.

19. Give an example of when you have called in sick to work even though you were not sick and why.

20. What is a job that you have been fired from, or otherwise lost the job?

21. What are things (is something) that make(s) you really happy?

22. How is your relationship with your father?

23. Who is someone you have kept your promise to?

24. How do you handle project deadlines?

25. Have you ever bragged about an accomplishment that you did not actually do yourself?

26. Do you think you sometimes drink too much?

27. Have you ever received a poor work performance evaluation at any job?

28. Do you usually make rash decisions or do you consider every aspect about it?

29. Have you ever sold something for more money than the item was actually worth?

30. Have you ever lied to a man/woman to try to get them to go out with you?

31. Have you ever lied on your resume?

32. Have you ever told an employer that you know how to do something when in fact you do not?

33. Have you ever run away from your parent’s house?

34. Have you deliberately lied to any of these questions?

35. Do you think you are a good driver?

36. Do you like reading? What is your favorite book?

Supplementary Figures

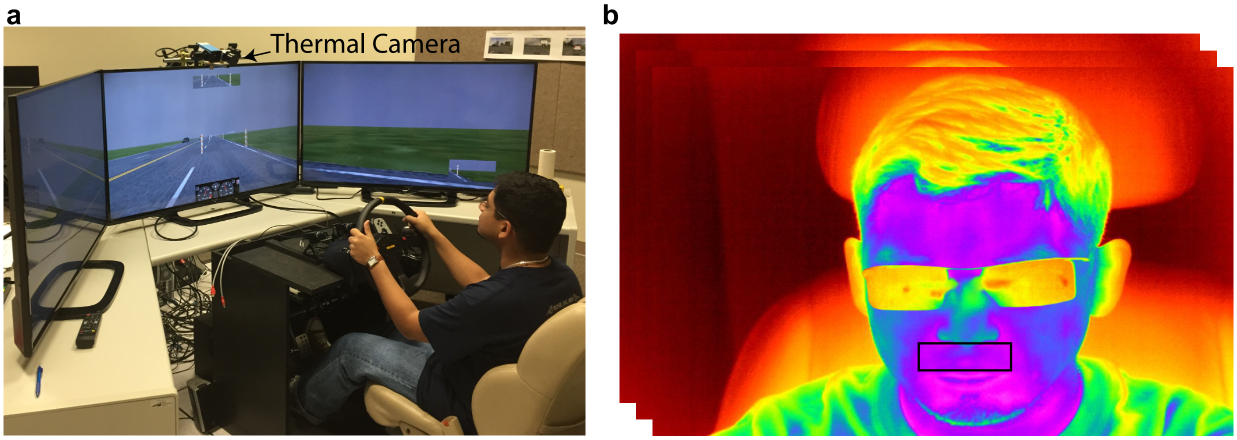

Supplement - Figure S1: **a,** Experimental set-up. The thermal camera is Tau 640 (FLIR Commercial Systems, Goleta, CA); it features a small size $(44 \times 44 \times 30$ mm), affordable price $(< \$5,000)$, and adequate thermal $(< 50$ mK) and spatial resolution (640×512). **b,** Corresponding facial thermal sequence. The back rectangle on the subject's lower perinasal area is the tracker[14], supporting perspiratory signal acquisition in the presence of natural head motion.

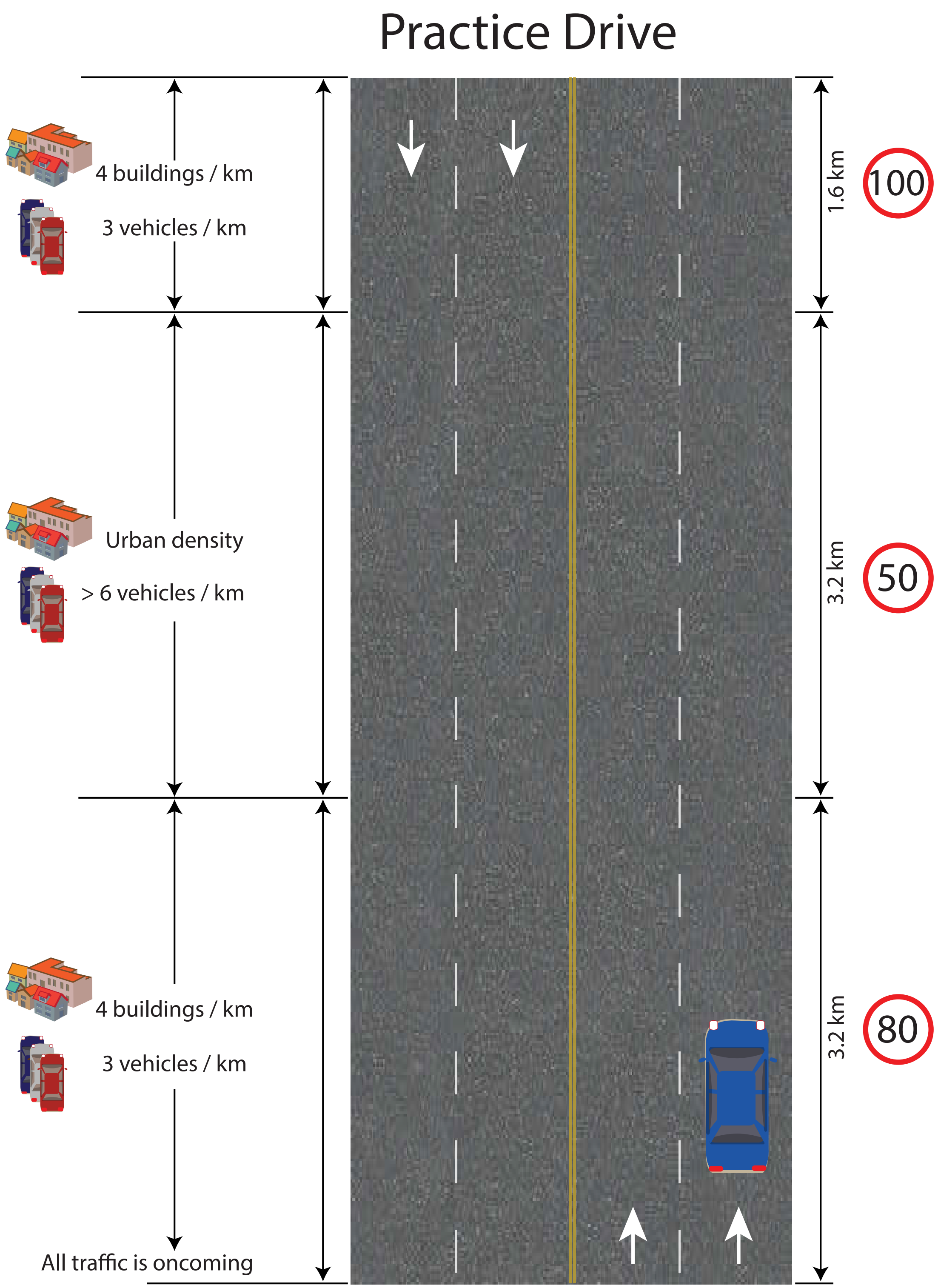

Supplement - Figure S2: Practice Drive (PD).

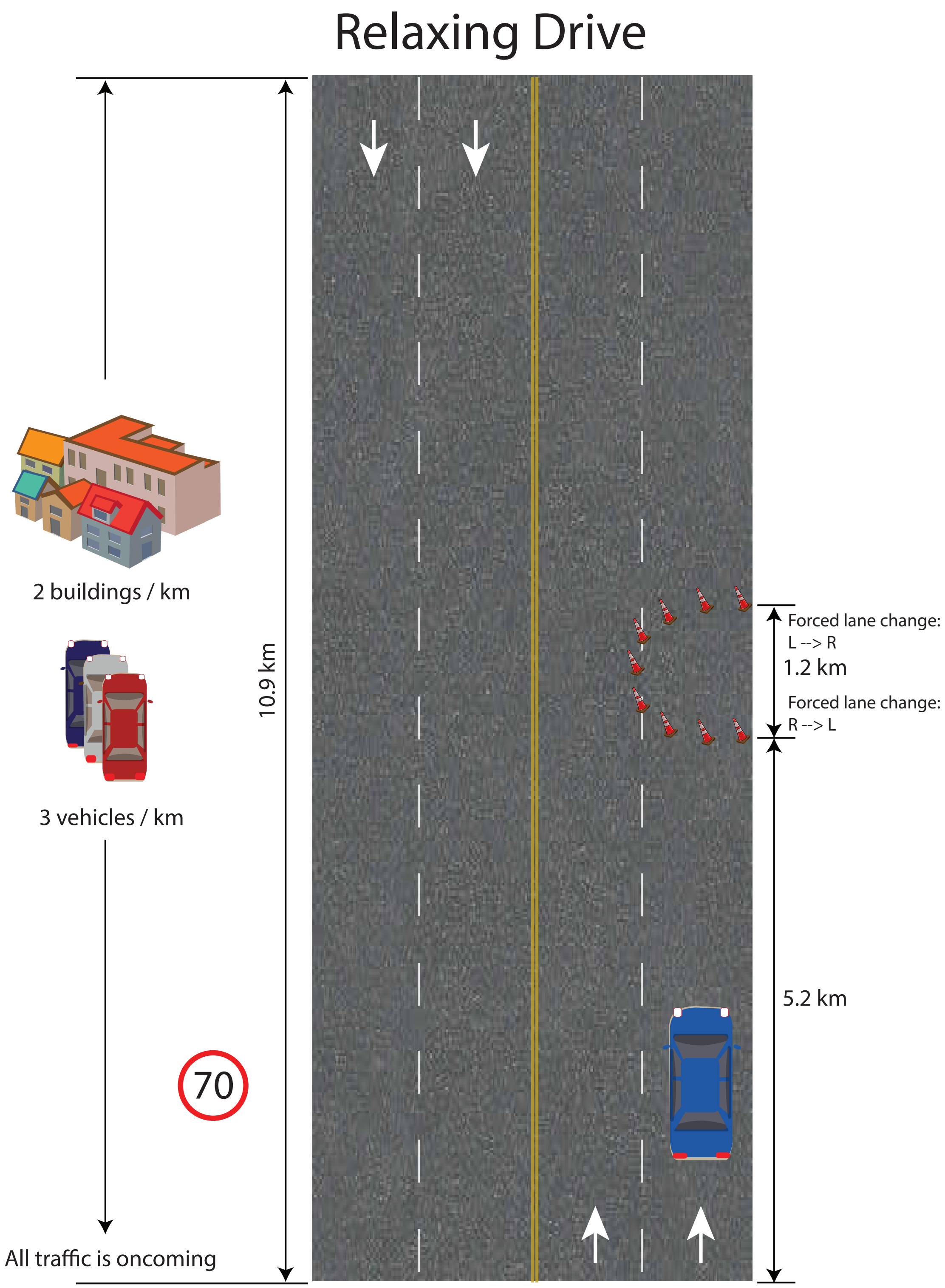

Supplement - Figure S3: Relaxing Drive (RD).

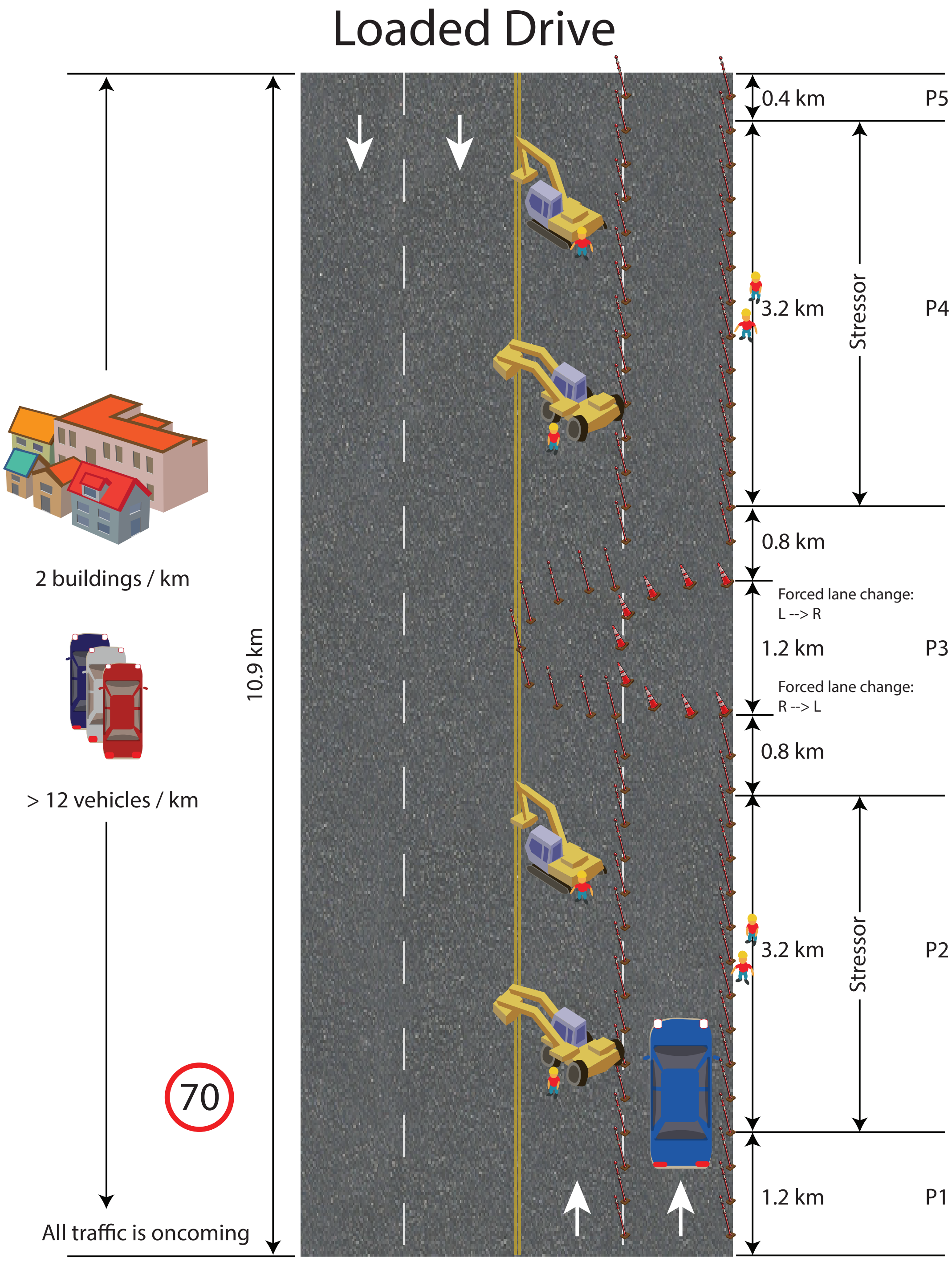

Supplement - Figure S4: Loaded Drives (LD).

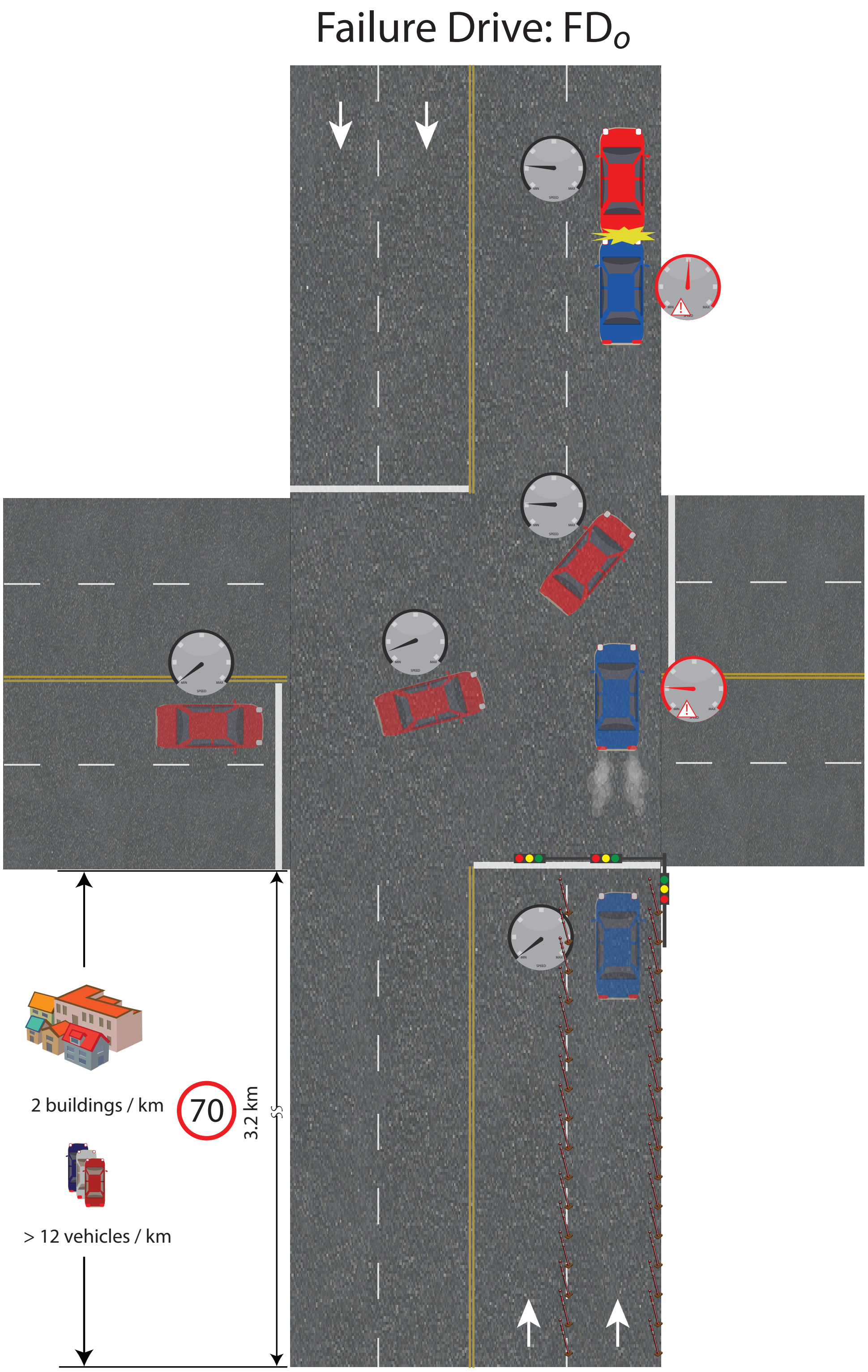

Supplement - Figure S5: Failure Drive (FD_o) - experimental arm $y = o$.

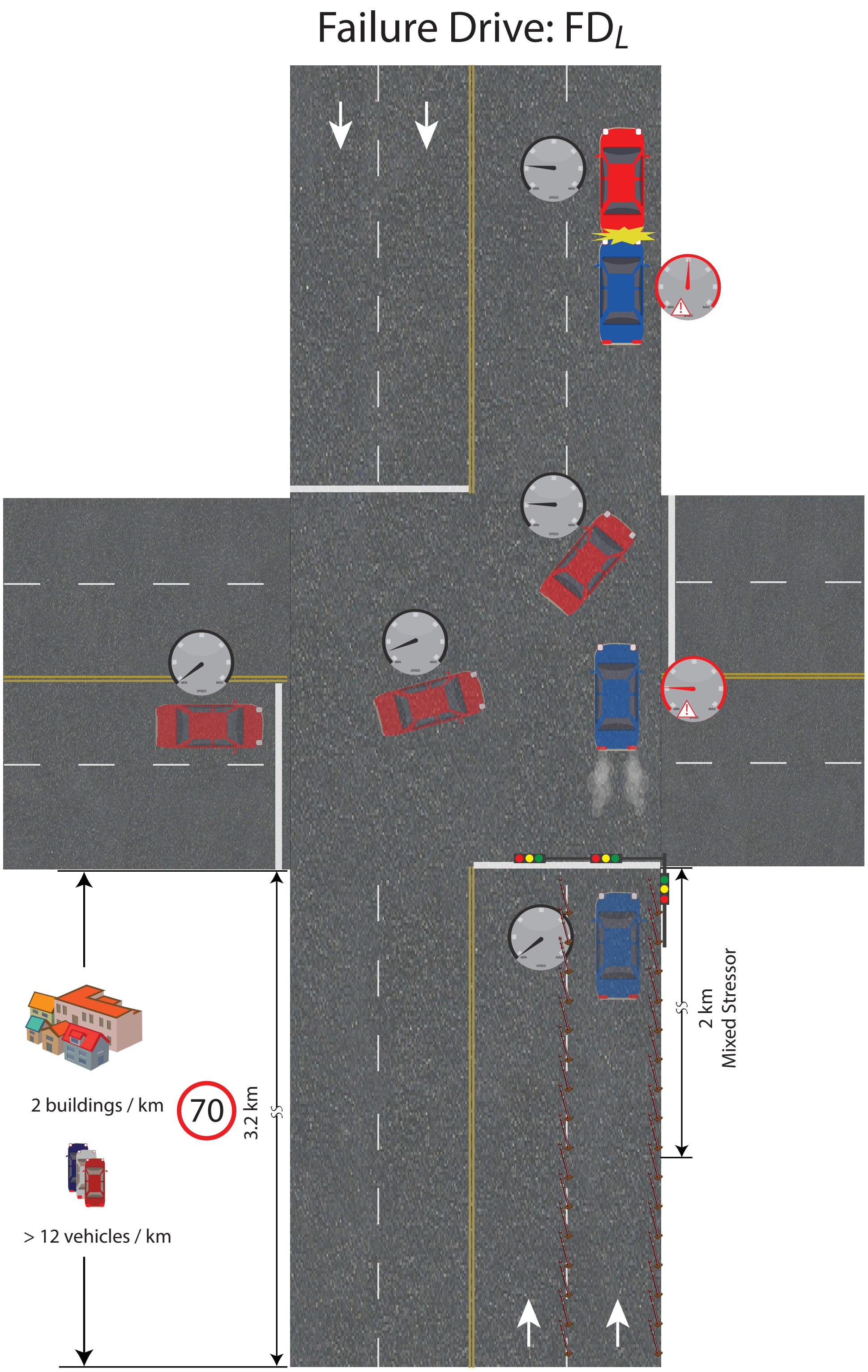

Supplement - Figure S6: Failure Drive (FD_L) - experimental arm $y = L$.

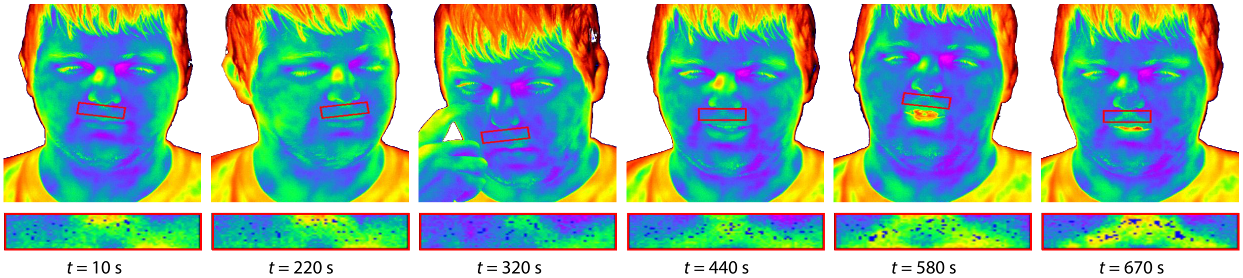

Supplement - Figure S7: **Top Thermal Image Row:** Perinasal tissue tracker (red rectangle) at work as Subject T013 exhibits head motion during driving in LD_E. **Bottom Thermal Image Row:** Motion-corrected perinasal area snapshots corresponding to the top row images. Proliferation of perspiration (dark spots), manifesting arousal, is evident towards the end of the sequence.
